# Knowledge, Beliefs and Practices of Patients with Diabetic Retinopathy at the University Hospital of the West Indies, Jamaica

**DOI:** 10.1007/s10900-015-0133-y

**Published:** 2015-12-18

**Authors:** Tecah Foster, Lizette Mowatt, Jasneth Mullings

**Affiliations:** University Hospital of the West Indies (UHWI), Kingston 7, Jamaica, West Indies; Department of Surgery, Faculty of Medical Sciences, University of the West Indies (UWI), Mona, Kingston 7, Jamaica, West Indies; Office of the Dean, Faculty of Medical Sciences, University of the West Indies, Mona, Jamaica, West Indies

**Keywords:** Knowledge, Beliefs, Practices, Diabetic retinopathy, Jamaica

## Abstract

To determine the knowledge, beliefs and practices of patients with diabetic retinopathy attending the Retina Eye Clinic at the University Hospital of the West Indies. A prospective study was done using a questionnaire with a sample population of 150 patients. The questions included their knowledge about the frequency of their eye examination, the relevance of exercise and a healthy diet, the role of the ophthalmologist and their views on the importance of compliance with medications for diabetes and hypertension. One hundred and fifty patients were recruited. Sixty six percent (99/150) were females and 34 % (51/150) males. The ages ranged from 29 to 83 years (mean ± SD, 56.1 ± 10.3) years. Type II diabetes was more common; 63 and 79 % of females and males respectively. A minority (19.8 %) obtained tertiary education. The mean % knowledge scores were 86 ± 14 for males and 82.8 ± 16.4 for females (*p* = 0.260). Prior to attending the retina clinic, 50 % were unaware of the need for annual eye examinations. Compliance with medication, exercise and a special diet was seen in 73, 40.3 and 49.7 % respectively. Current knowledge scores were good. However, knowledge about the timing and frequency of eye examinations prior to attending the retina clinic was inadequate. Correct knowledge and beliefs did not correspond to a high level of compliant practices.

## Introduction

There are ~387 million people worldwide with diabetes [1]. Diabetic retinopathy (DR) is the leading cause of blindness among the working age population [2]. In Europe, DR is the cause of blindness in 30–50 % of type 2 diabetes mellitus (DM) patients [3]. In North America and the Caribbean the prevalence of diabetes is ~11.4 % with 27.1 % of the population undiagnosed [1]. The prevalence in older adults (>60 years old) in Jamaica (*total pop. 2.7 million*) is 26 %, which represents a 157 % increase over 23 years [4]. This potentially makes DR an increasing public health challenge in our country. Despite its prevalence and its sight threatening potential, DR is a preventable cause of blindness.

Patient’s knowledge of DM effects on the eye varies geographically [5–7]. A Pennsylvania USA study on low income DM women, showed that >50 % of 150 patients were unaware that DR may be asymptomatic and preventable. The type of health provider who should perform an eye exam was unknown in 20 % of cases and 17 % did not know that annual eye exams were recommended [5]. Over 75 % were unaware that a dilated eye examination should be done [5].

In contrast, 72.9 % of patients had excellent knowledge of eye complications, with 52 % having an excellent practice of periodic eye checks in Oman [6]. In Australia and Japan, 96 and 98 % of patients respectively had an excellent knowledge of eye complications [6]. High literacy rates and proactive counselling were considered the reasons. However, in a New York survey, 35 % of patients with diabetes did not follow the vision care guidelines; 66 % were not having annual eye examinations and 33 % had undilated eye examinations [7].

The purpose of this study was to assess the knowledge, beliefs and practices of patients with DR attending the Retina Clinic at the University Hospital of the West Indies (UHWI) in Kingston Jamaica. Although patients with DR are screened and treated at various public and private settings islandwide, UHWI, a type A hospital, has the only Specialist Retina Clinic in the island.

Anticipated benefits of this study included gaining insight into the issues involved in addressing the problem of DR. This could further guide the implementation of strategies to reduce the prevalence and severity of DR in Jamaica.

## Subjects and Methods

A prospective cross sectional study was designed to assess the knowledge, beliefs and practices of patients with DM at the Retina Clinic, UHWI. One hundred and fifty adult patients were recruited consecutively from the retina clinic over a 6 month period. Inclusion criteria were adult patients (≥18 years and older) with DR attending the retina clinic at UHWI. Exclusion criteria were patients unable to comprehend questions or communicate effectively, hearing impaired, patients with other ocular comorbidities and past history of eye trauma. Persons who met the criteria were invited to participate.

**Fig. 1:**
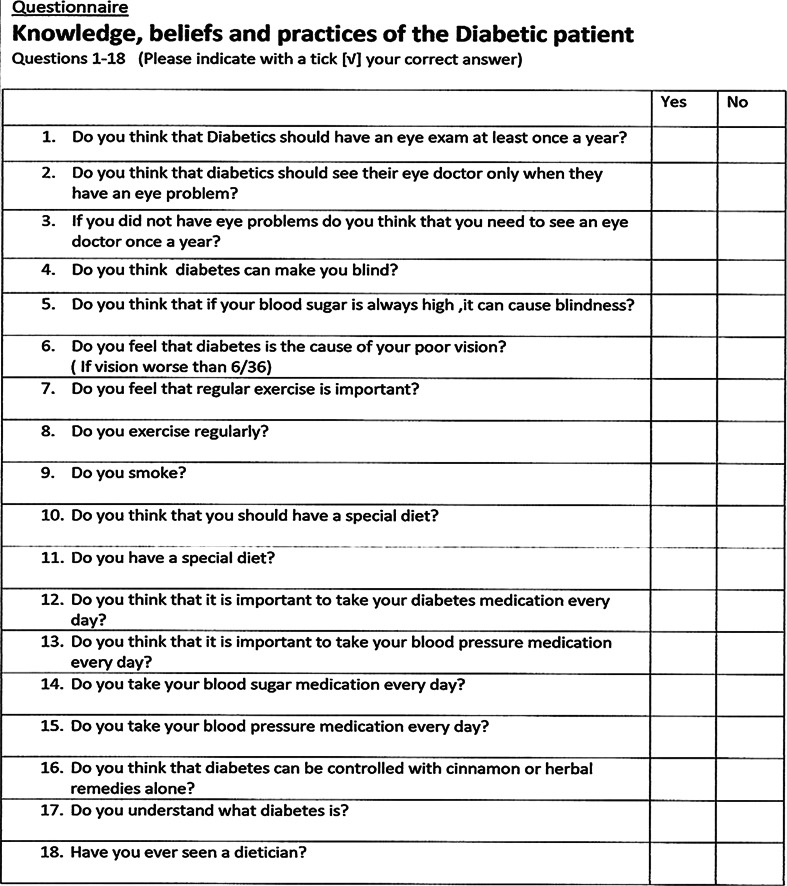

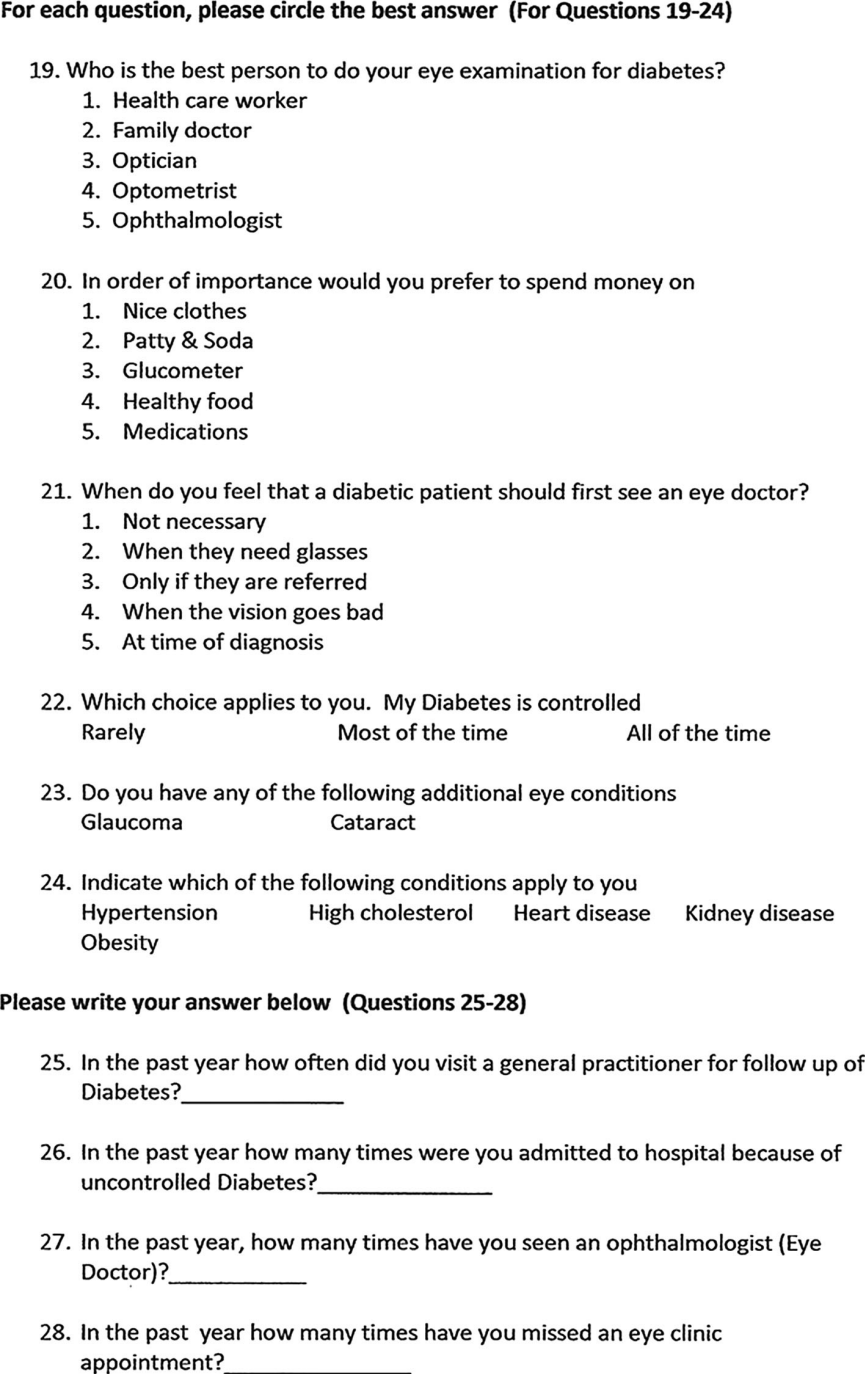
Questionnaire on knowledge, beliefs and practices

Informed voluntary written consent was obtained. Patients were individually interviewed by the same person using a questionnaire that had 28 questions. Data were collected on age, gender, level of education, and average monthly income. The study investigated their knowledge of diabetes, health seeking behaviours such as frequency of their eye examination, lifestyle practices including exercise and a healthy diet, smoking and compliance with medications, exercise and a special diet, and attitudes towards the management of their condition, the role of the ophthalmologist, as well as the importance of compliance with medications, exercise and a special diet (Fig. 1a, b).

Measurements collected included weight, height, Body Mass Index (BMI), blood pressure (BP) and HbA1c. Patients were weighed on a balanced beam scale in kg (±0.1 kg) and their heights measured on a stadiometer in meters (±0.1 m) to assess their BMI. Blood pressure was checked with a sphygmomanometer (±1 mmHg) and Snellen visual acuity assessed. Blood samples were taken to determine glycosylated haemoglobin levels (HbA1c) using a BIO-RAD D-10 analyser.

Data was analysed using SPSS software version 19. Descriptive analyses were performed using Chi square test for categorical variables and the *t* test for continuous variables. Statistical significance was accepted at *p* < 0.05 Chi square tests.

Confidentiality and anonymity were ensured. The research protocol was approved by the Ethics committee, Faculty of Medical Sciences at the University of the West Indies.

## Results

One hundred and fifty patients, 51 males (34 %) and 99 females (66 %) were recruited. The age range for males was 47.3–66.1 years (mean 56.7 ± 9.4). The age range for females was 45.3–68.7 years (mean 57 ± 11.7 years).

### Type of Diabetes Mellitus (DM)

**Fig. 2:**
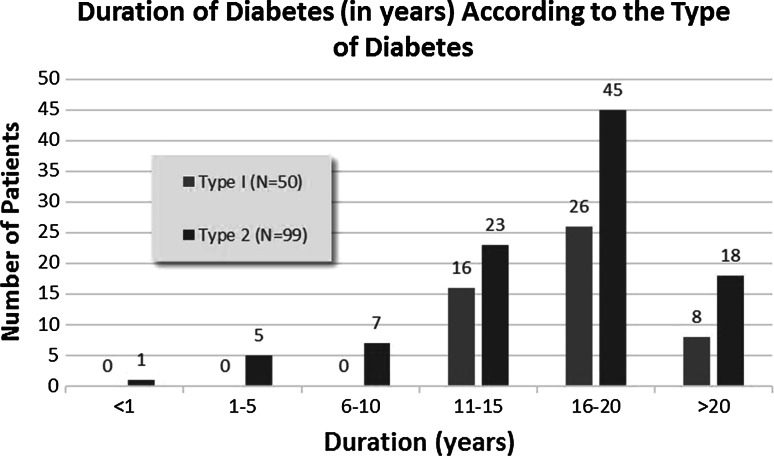
Duration of diabetes mellitus according to type of diabetes

The ratio of type 1 to type 2 DM was 1:3.8 for males and 1:1.7 for females. Type 2 DM was more common in the males (73 %) than females (62 %; *p* = 0.206). One male was unable to recall the duration of his diabetes. The majority (85 %) had DM for >10 years (Fig. 2).

### Educational Level, Employment and Income

Only 18.9 % had tertiary education as their highest level of education with 36.2 and 44.3 % having secondary and primary education respectively. Primary education was achieved in 50 and 44.4 % of the males and females respectively. However, 40.4 and 15.1 % of females compared with 29.9 and 24.1 % of males achieved secondary and tertiary level education respectively. There was no significant difference in the level of education between males and females (*p* = 0.226).

The majority of patients (73 %) were unemployed. Of the employed patients, 62.5 % earned an income ≤J$50,000 (US$446) per month and 37.5 % earned ≥J$50,000 ($US446) per month.

### Body Mass Index (BMI)

**Fig. 3:**
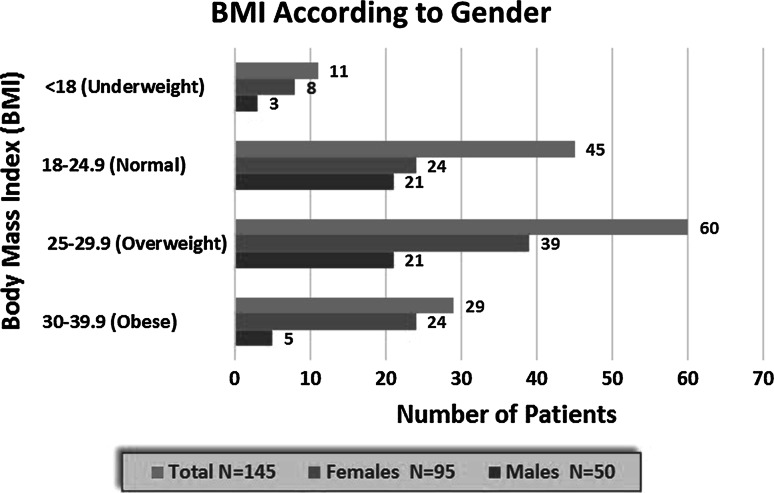
Distribution of body mass index according to gender

The majority of patients (61.4 %) were overweight with a BMI value >25 kg/m^2^ (Fig. 3). The range of BMI for males was 17.9–31.1 kg/m^2^ (mean 24.5 ± 6.6). Females had a BMI range of 17.7–34.1 (mean 25.9 ± 8.2). The BMI could not be calculated in 5 patients (4 females and 1 male) because their weight and height could not be accurately measured, as they were either wheel chair bound, and or could not stand up erect for measurement. The difference in overweight status between males (62 %) and females (66.4 %), was not significant (*p* = 0.077).

#### Body Mass Index According to Type of DM

In the type 1 DM patients, the males (22.6 ± 11.4) had a lower BMI compared to females (25.3 ± 10.6). In type 2 DM patients, the males (25.2 ± 3.5) also had a lower BMI compared to females (26.2 ± 6.4). However the BMI for the type 2 DM patients were higher than the Type 1 DM. There was no significant difference in mean BMI according to type of DM (*p* = 0.353).

### Glycosylated Haemoglobin *(*HbA1C*)*

The mean HbA1c values for the study group were 7.7 ± 2.0 % and 8.6 ± 2.1 % for males and females respectively (*p* = 0.05). The mean HbA1c values for persons with type 1 and type 2 DM were 8.6 ± 2.3 % and 8 ± 1.9 respectively (*p* = 0.626). Males had significantly better HbA1c levels, with 45.2 % of males versus 15.9 % of females with HbA1c values ≤6.5 % (*p* = 0.005).

### Blood Pressure (BP)

The systolic BP range for the females was 127–168 mm Hg (mean 147.2 ± 20.4), whilst that for the males was 131–174 mm Hg (mean 152 ± 21.5) (*p* = 0.239). The diastolic BP range for the females was 75–95 mm Hg (mean 84.6 ± 10.3) and that for the males was 78–97 mm Hg (mean 87.3 ± 9.4) (*p* = 0.122). There were no significant differences between the systolic and diastolic blood pressure readings for males and females.

### Visual Acuity

The Snellen visual acuity was converted to LogMAR for statistical analysis. Mean Log Mar visual acuity was 0.9 ± 0.98 and 0.67 ± 0.80 for the right and left eye respectively (*p* = 0.03). LogMar visual acuity for the left eye was worse in males (*p* = 0.049). There was no significant sex difference for the logMar visual acuity in the right eye (*p* = 0.47).

### Knowledge Scores

The overall mean knowledge score’s percentages were 86 ± 14 % for males and 82.8 ± 16.4 % for females *p* = 0.260 (Table 1). The association between knowledge and education level approached significance (*F* = 2.293; *p* = 0.08). There was no significant relationship between knowledge and duration of DM (*p* = 0.111). No significant relationships were found with good glucose control, HbA1c levels and the level of knowledge (*p* = 0.028).

**Table 1:** Comparison of the Questionnaire’s correct responses by gender

Question #	Percentage of correct responses (%)		*p* value
	Males (N = 51)	Females (N = 99)	
1	98.1	95.8	0.423
2	84.6	94.8	**0.035**
3	88.2	88.7	0.568
4	96.2	97	0.413
5	98.1	96.9	0.560
6	88.2	78.7	**0.039**
7	100	98.9	0.651
8	36.5	42.3	0.308
9	96.2	94.8	0.534
10	100	93.8	0.075
11	44.2	52.6	0.212
12	63.5	78.9	**0.042**
13	84.6	73.2	0.075
14	63.5	78.9	**0.042**
15	62.5	75.7	0.088
16	71.4	73.3	0.480
17	61.5	53.6	0.225
18	61.5	68.0	0.268

Figure 1a, b show the questionnaire

*p* values < 0.05 are highlighted in bold

When patients were asked to relate their understanding of diabetes (question # 17), 43 % of patients were unable to accurately explain. The majority (82 %) of patients were aware that screening for DR should be done at the time of diagnosis. However 60 % of them were not aware of this prior to being referred to the Retina Clinic. The ophthalmologist was stated as the ideal person for their eye examinations in 60 % of cases. In our study, 97 % of patients were aware of the need for annual DR screening but 50 % of these persons were unaware of this prior to their referral.

Questions with responses having significant (*p* ≤ 0.05) gender differences are listed below.

*2. Do you think that diabetics should see their eye doctor only when they have an eye problem?
*6. Do you feel that diabetes is the cause of your visual impairment?
*12. Do you think that it is important to take your diabetes medication every day?
*14. Do you take your blood sugar medication every day?

#### Knowledge According to Gender and Type of DM

Females were more aware of visiting their eye doctor, compliance with medications and blood testing (Table 1). With regards to an analysis according to the type of DM, only one knowledge based question (question # 2) showed a significant difference. Most type 1 DM (98 %) versus 83.5 % of type 2 DM patients thought that annual eye examinations were important (*p* = 0.008).

### Beliefs

Overall the majority of patients had healthy beliefs with 95.3 % of patients believing that it was important to take their medication for diabetes regularly. Regular exercise was deemed important in 97.3 % of patients. Whereas, 95.9 % believed that a special diet was important.

#### Beliefs Versus Practice

**Fig. 4:**
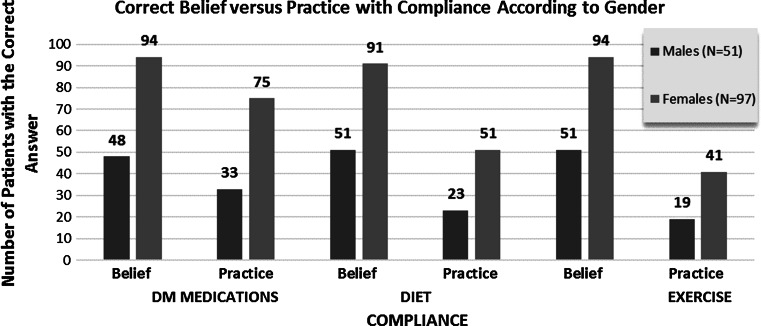
Comparison of correct beliefs about compliance and actual practice according to gender

Belief versus practice with regards to medication, exercise and a special diet is shown in Fig. 4. Compliance with medication was the belief in 95.3 % of patients, however, 73 % practised compliance (*p* = 0.005). Likewise, 97.3 % of patients believed that regular exercise was important however, only 40.3 % had a regular exercise routine (*p* = 0.06). The importance of a special diet was seen in 95.9 % of patients and 65.8 % had seen a dietician, however only 49.7 % practiced having a special diet (*p* = 0.111). Most patients (96 %) were non smokers.

## Discussion

According to the International Diabetes Federation, diabetes prevalence is highest in resource-limited settings; with 80 % of people with diabetes globally, living in low and middle income countries and the greatest number of patients with type 2 diabetes being between 40 and 59 years old [1]. These findings were reflected in this sample of 150 patients as 73 % were unemployed and the majority of those employed had low income, 62.9 % had type 2 diabetes and the mean age was 56.9 ± 10.9 years.

A major non-modifiable determinant of the development and progression of DR is duration of diabetes [8]. Since the majority of patients in this study (85 %) had a duration of diabetes of ≥10 years they were already at an increased risk for progression of DR.

The ratio of males to females with DR was 1:1.9. Diabetes is more common in Jamaican females [9, 10]. Thus the ratio can either be explained by a greater number of females in the Jamaican population with diabetes and DR or a greater proportion of males in the population who are either undiagnosed with diabetes or DR.

Gender had no significant association with age, type of DM nor duration of DM. However the relationships between gender and HbA1c and LogMAR visual acuity (left eye) were significant (0.05 and 0.049 respectively), while the gender and BMI relationship approached significance (*p* = 0.07).

In Jamaica, obesity was found to be more prevalent in females compared to males (30 % compared to 10 %) [11]. Our females had higher BMI measurements than males (*p* = 0.077), which may be due to cultural acceptance of obesity in females [9]. Other reasons could include a sedentary lifestyle, lacking exercise or difficulties with maintaining a special diet among females.

Zimmet et al. [12] reported that changes in human behavior and lifestyle such as a sedentary lifestyle, unhealthy nutrition and obesity resulted in a dramatic increase in the incidence of type 2 diabetes worldwide. The abandonment of healthier traditional lifestyles such as a regular exercise routine largely as a result of dependence on motorized transport have also had a negative effect on the health of patients with diabetes. These lifestyles have contributed to a rise in levels of obesity and overweight in the population increasing the risk for diabetes [13]. The lack of a special diet and exercise routine was seen among patients in this sample. The significantly higher HbA1c levels for females than males (*p* = 0.05) could be a result of the higher BMI measurements since obesity is a risk factor for uncontrolled diabetes.

One study considered a knowledge score of ≥80 % as adequate [9] while in another study that percentage was considered between a moderate and good knowledge score [13]. In this study a score ≥80 % in the knowledge section was considered good, 60 to <80 % moderate, 50 to <60 % as fair and <50 % as poor.

Both genders demonstrated good current knowledge based on the mean knowledge scores. The only knowledge based question that showed a significant difference with gender was question # 2. Five point two percent of females and 15.4 % of males felt that they should see an Ophthalmologist only when they have visual impairment (*p* = 0.035). This could provide a possible explanation for a smaller percentage of males in the sample as perhaps there are more undiagnosed cases of DR among men than women since males may wait for visual impairment before having an eye examination.

Since 43 % of persons were unable to explain DM, many patients were unaware of the processes involved in the disease and the potential seriousness of uncontrolled diabetes. This may suggest that health education is being done as a monologue instead of a dialogue which would involve the patient explaining in their own words their understanding of diabetes and potential consequences for them. Since there was a positive correlation between knowledge and educational level those with lower educational level may need to be given more attention or be educated about diabetes in simple or “layman’s” terms that they can understand. One educational intervention study in adults with diabetes in Erode District South India documented an improvement in KAP score [14].

Males had worse vision than females (*p* = 0.049). A factor that could contribute to this include the findings that less males than females were compliant with taking medication for DM (*p* = 0.04).

The significantly greater proportion of type 2 DM patients with DR could be explained by the fact that more type 1 DM patients thought that annual eye examinations were important even if there was no visual impairment (*p* = 0.008).

There were deficiencies in early knowledge about DR screening which could have aided earlier diagnosis and less progression, prior to referral to the retina clinic. DR screening should be done early from the time of diagnosis of diabetes. Early knowledge about the importance of lifestyle modification is also necessary. Patients with diabetes need to know that DR in its early stage can be asymptomatic and so they should not wait until they have visual impairment before getting an early dilated fundus examination at the time of diagnosis. The awareness that early detection and treatment can halt progression of DR and save their vision and functional capacity needs to be promoted.

More patients need to be advised that the most experienced person to perform a dilated eye exam for DR is the ophthalmologist and not the optician, optometrist or family doctor. This could prevent late presentation to the Ophthalmologist and thereby, preventing visual loss. Alternatively establishing a DR screening program in Jamaica with health care personnel trained at grading DR and referral when necessary could be very helpful.

Despite the importance of knowledge, this study showed that having the necessary knowledge can be insufficient to implement a healthy lifestyle and prevent progression of DR. Based on the correct beliefs versus practices data more patients need to practice their correct beliefs.

Financial support to implement healthy measures such as having and sustaining a healthy but costly diet and a gym membership may be a factor. Lack of financial support was a common complaint stated by patients. This was likely due to the fact that the majority of patients (73 %) were unemployed and most of those employed had a low income. This could partly explain the reason for the finding of healthy beliefs not corresponding to healthy practices even though the level of knowledge at the time of the interview was high. Perhaps education about low budget means of supporting a healthy diet and exercise regime, for example, a home or community vegetable garden and walking reasonable distances instead of taking public transportation would be relevant for such patients.

Policy measures to assist and encourage patients to have a healthy diet and a regular exercise routine need to be developed and implemented. Obesity and overweight patients made up 71.4 % of our study population. The World Health Organization has recognized that there is a global epidemic of obesity associated with the rising prevalence of type 2 DM [15]. One study in Karachi revealed that 69.9 % of patients with diabetes in the group did not exercise and 49 % took high caloric snacks between meals [16]. Since exercise plays a key role in regulating blood glucose levels, the absence of a regular exercise routine in our DM patients interviewed was significant.

Financial constraints, feeling unsafe while walking, fear of injury while walking due to poor vision and the absence of energy and motivation to exercise were among the reasons stated for not having a regular exercise routine. These concerns could be addressed by public health and community interventions, with collaborative efforts of public and private institutions. Discounted gym memberships for patients with diabetes, more free secure parks for walking in both urban and rural communities, and exercise support groups with trained instructors who could teach exercise routines that are safe for those with poor vision are some possible measures that could help. The lack of energy and motivation to exercise as a barrier to implementing a regular exercise routine could suggest that some of these patients suffer from depression.

Some participants confessed to feeling stressed and overwhelmed by the visual impairment and other burdens of diabetes. Unfortunately diabetes-specific emotional stress has been associated with poor glycemic control *p* = 0.007 [17]. Also having diabetes has been found to double the odds of comorbid depression (OR = 2.0, 95 % CI 1.8–2.2) [18].

Psychologists, psychiatrists and counsellors could therefore contribute to the multidisciplinary management of diabetes by screening for depression and providing counselling on coping mechanisms and stress management for patients and their families. Medical doctors can also be sensitive to the need for referring patients when necessary to seek such help. One study showed 97 % of family physicians will give lifestyle counselling and refer patients to a dietician. However, only 43 % of physicians recommended group support meetings, which may be more helpful to encourage sustained good practice [19].

With respect to the lack of a special diet, healthy eating could be supported by popular fast food chains placing nutritionally balanced options on their menu. Also patients with diabetes could be granted discounts on purchasing healthy food or offered plots of land or financial support to support community vegetable gardens. Also the nutritional value and sugar content should be more available and visible on food in fast food places and supermarkets.

Some similar societal related barriers found in a KAP study conducted among patients with diabetes in Barbados included high cost and limited availability of appropriate food and availability of exercise facilities [20].

Compliance with medication for diabetes was adequate (*p* = 0.005). In Jamaica the National Health Fund (NHF) has been recognized for their efforts in making medication for chronic illnesses like diabetes more affordable. This could have contributed to the adequate compliance seen in this study. However 24.7 % of patients confessed to poor compliance with medication. Reasons such as undesirable side effects, long waiting lines at pharmacies, lack of family support to access the medication and again financial constraints were found. Similarly in the previously mentioned KAP study done among patients with diabetes in Barbados health care factors that had a negative impact on management of diabetes included the long waiting times in public sector clinics and pharmacies [20].

Although the sample size of 150 patients was appropriate based on the population size, completing the recruitment of patients in a shorter time was limited by a number of factors. Firstly many patients with diabetes attending the UHWI retina clinic had ocular comorbidities that were included in the exclusion criteria. Many patients had primary open angle glaucoma among other sight threatening comorbidities that would have been conflicting in terms of contributing to visual impairment. Another factor was patients who were lost to immediate referral to the accident and emergency department because of dangerously elevated blood glucose and blood pressure readings. This subset of patients would have been an important group as they could likely have had significant DR considering their poor control.

Additionally some patients did not wish to give consent either because of feeling unwell or not being in a receptive mood for an interview. In order to avoid bias the same interviewer was used for the study. All blood samples for HbA1c testing was done at a single laboratory, to prevent inconsistencies with method of testing.

A significant relationship was not found between HbA1c and the level of knowledge. This maybe because having a high level of knowledge was not proven to correspond to all patients having a healthy behavior and thus better control of diabetes. Therefore, knowledge and beliefs were not well correlated with practices.

Current knowledge and beliefs was good. However, knowledge about the timing and frequency of eye examinations for DR screening prior to being referred to the retina clinic was inadequate. This is important as it allows DR to be detected in a timely manner that will allow early treatment and prevention of visual loss. Correct knowledge and beliefs did not always correspond to compliant practices primarily with respect to exercise and having a special diet. Therefore, although correct knowledge is important other factors influence compliance. Health practitioners must implement effective methods of helping patients to translate their knowledge into appropriate practices.
